# An investigation of stigmatizing attitudes towards people living with HIV/AIDS by doctors and nurses in Vientiane, Lao PDR

**DOI:** 10.1186/s12913-017-2068-8

**Published:** 2017-02-10

**Authors:** Savina Vorasane, Masamine Jimba, Kimiyo Kikuchi, Junko Yasuoka, Keiko Nanishi, Jo Durham, Vanphanom Sychareun

**Affiliations:** 1 Department of Radiology, Mahosoth hospital, Vientiane, Lao PDR; 2 Department of Community and Global Health, Graduate School of Medicine, The University of Tokyo, Tokyo, Japan; 3 School of Public Health, The University of Queensland , Brisbane, Australia; 4 Dean of the Faculty of Postgraduate Studies, University of Health Sciences, Ministry of Health, Vientiane, Lao PDR

**Keywords:** HIV-related stigma, Discrimination, Healthcare workers, Lao PDR

## Abstract

**Background:**

Despite global efforts, HIV-related stigma continues to negatively impact the health and well-being of people living with HIV/AIDS. Even in healthcare settings, people with HIV/AIDS experience discrimination. Anecdotal evidence suggests that healthcare professionals in the Lao People's Democratic Republic, a lower-middle income country situated in Southeast Asia, stigmatize HIV/AID patients. The purpose of this study was to assess HIV stigmatizing attitudes within Laotian healthcare service providers and examine some of the factors associated with HIV/AIDS-related stigma among doctors and nurses.

**Methods:**

A structured questionnaire, which included a HIV-related stigma scale consisting of 17 items, was self-completed by 558 healthcare workers from 12 of the 17 hospitals in Vientiane. Five hospitals were excluded because they had less than 10 staff and these staff were not always present. The questionnaire was pre-tested with 40 healthcare workers. Descriptive statistical analysis was performed and comparisons between groups undertaken using chi-square test and *t*-test. Bivariate and multiple linear regression analyses were carried out to examine the associations between stigmatizing attitudes and independent variables.

**Results:**

Out of the 558 participating healthcare workers, 277 (49.7%) were doctors and 281 (50.3%) were nurses. Nearly 50% of doctors and nurses included in the study had high levels of stigmatizing attitudes towards people living with HIV/AIDS. Across the different health professionals included in this study, lower levels of HIV/AIDS knowledge were associated with higher levels of stigmatizing attitudes towards people living with HIV/AIDS. Stigmatizing attitudes, including discrimination at work, fear of AIDS, and prejudice, were lower in healthcare workers with more experience in treating HIV/AIDS patients.

**Conclusions:**

This study is the first to report on HIV/AIDS-related stigmatization among healthcare workers in Lao PDR. Stigmatizing attitudes contribute to missed opportunities for prevention, education and treatment, undermining efforts to manage and prevent HIV. Reversing stigmatizing attitudes and practices requires interventions that address affective, cognitive and behavioral aspects of stigma. Alongside this, health professionals need to be enabled to enact universal precautions and prevent occupational transmission of HIV.

**Electronic supplementary material:**

The online version of this article (doi:10.1186/s12913-017-2068-8) contains supplementary material, which is available to authorized users.

## Background

Despite global progress in the treatment and care of HIV positive individuals and community education, HIV-related stigma and discrimination continues to prevent people from accessing HIV testing, treatment and care [1–9]. Stigma in the healthcare sector has often been found to be particularly pernicious, and a contributor to poor health outcomes [9–11]. There are at least three major pathways to HIV stigma within healthcare facilities [10], namely the fear of contracting HIV, not being aware of potentially stigmatizing attitudes and behaviors and the impact of stigma, and associating HIV with immoral behavior [10]. Aside from access to services, other critical reasons for reducing HIV/AIDS-related stigma is the negative affect stigma has on a person’s self-concept and mental health [12–14], life satisfaction [15], and quality of life [15, 16].

A commonly used definition of stigma in the HIV/AIDS literature is “prejudice, discounting, discrediting, and discrimination directed at people perceived to have AIDS” ([17] p. 1107) and is informed by the work of Goffman [18]. It relates to the prejudicial feelings, stereotypical perceptions, discriminatory behaviors and actions, or social devaluation of HIV infection and related illnesses, as well as the activities associated with HIV-infection, and people living with HIV/AIDS (PLWHA) [19, 20]. It can be perceived and experienced, either internally or externally, by PLWHA. It can be enacted by those who are HIV-negative, including healthcare workers [8, 20, 21]. Stigma’s presence and its enactment are separate stigmatizing processes, where the devaluation of an attribute or trait contributes to negative beliefs. These beliefs are then enacted, creating distance either in perceived, or real, similarity from the devalued characteristic [22]. The ways in which stigma is enacted and perceived is also shaped by broader societal attitudes, and is thus dynamic and a result of both socio-cognitive and structural factors, created at the intersection of culture, power, and difference [20, 23, 24]. While similar to stigma, the focus of prejudice is on human characteristics, for example race or PLWHA, rather than deviant behavior and identities, disease and disabilities [25]. HIV prejudice, therefore, is a negative emotion, attitude or reaction towards PLWHA, and includes negative cognitive schemas or beliefs regarding PLWHA and can be partly explained by the fear that surrounds the disease. Discrimination towards PLWHA is the behavioral response of prejudice [8, 25] and can be understood in terms of social processes of power and domination with some groups, in this case PLWHA, that serve to devalue the stigmatized [21, 26].

High levels of HIV-related stigma have been identified in countries with lower levels of HIV prevalence and limited access to antiretroviral therapy (ART) [26–29]. At the individual level, HIV-related stigma has been associated with insufficient levels of HIV/AIDS knowledge [30], fear of casual transmission at the workplace [3, 26, 31] and low exposure to PLWHA [6, 30]. The purpose of this study was to assess HIV stigmatizing attitudes within healthcare service providers in the Lao People’s Democratic Republic (PDR) and examine some of the factors associated with HIV/AIDS-related stigma among doctors and nurses. Our overall intent was to inform policy discussions and develop effective interventions.

The Lao PDR has an estimated HIV prevalence of 0.3% among 15–49 years old and, based on the Asian Epidemic Model, is considered a low HIV prevalence country [32]. There have been few empirical studies that have examined and measured HIV-related stigma in healthcare workers in the Lao PDR. Anecdotal information, however, suggests that healthcare professionals in Lao PDR may engage in stigmatization of, and discrimination against, PLWHA [33, 34]. One report for example, noted that 12% of 84 PLWHA in Vientiane stated they had experienced stigmatizing attitudes from medical professionals [34]. Some medical workers have also been reported as trying to avoid or even refusing to care for PLWHA [34]. The practices and factors that are related to health worker discrimination towards HIV/AIDS patients in Lao PDR are, however, largely undocumented. Yet, understanding HIV-related stigma and discrimination in the Lao healthcare sector is particularly important, given the potential for increased transmission as the country rapidly integrates into regional and global markets [32]. This is characterized by increased migration, livelihood diversification and changing socio-cultural norms, and there are concerns that the potential exists for a generalized or concentrated HIV epidemic [32].

## Methods

A cross-sectional study was conducted in Vientiane, Lao PDR. Vientiane has nine districts, and was estimated to have a population of 797,130 in 2012 [35]. The dataset supporting the conclusions of this article is available in the Open Science Framework repository [osf.io/c6t4u].

### Study setting

**Fig. 1:**
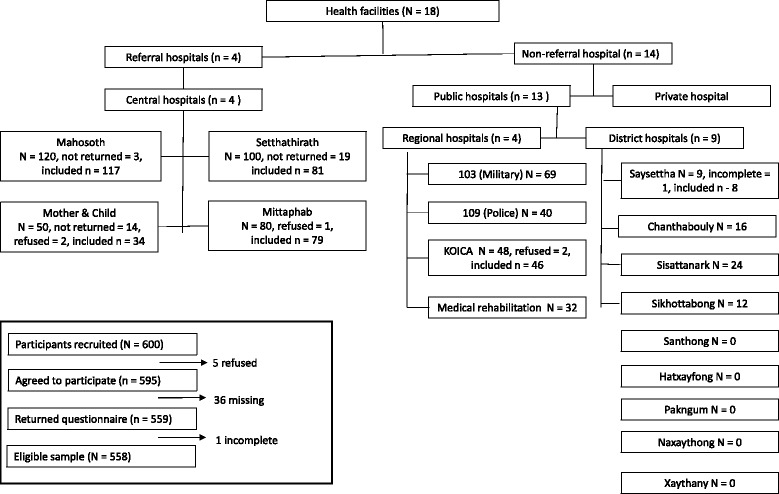
Study sampling procedures and final sample

The setting was public healthcare facilities in Vientiane, Lao PDR. Healthcare services in Lao PDR are primarily public, with the private health sector limited mainly to licensed pharmacies, clinics and informal providers [36]. The public healthcare facilities in Lao PDR consist of four central teaching (referral) hospitals, five regional hospitals, 13 provincial hospitals, 127 district hospitals, and approximately 746 health centers [36]. HIV/AIDS care wards are only in the referral (central) hospitals. Vientiane has four central teaching hospitals, four regional hospitals, nine district hospitals, and 213 health centers. From the nine districts in Vientiane, a convenience sampling method was used, with four districts (Saysettha, Chanthabouly, Sisattanak and Sikhottabong) selected. This yielded a sample of four central (referral) hospitals, four regional (non-referral) hospitals and four district hospitals for the study, making a total of 12 out of a possible 17 hospitals being selected (see Fig. 1). The other five district hospitals were excluded because they either had less than 10 healthcare workers or staff were not always present.

### Study population and sampling

There are 14,189 public sector health workers, with Ministry of Health staff and other health staff from the ministries of National Security and National Defense making up approximately 30% of total health work force [37]. The majority of doctors and nurses work in Vientiane. All doctors and nurses who were employed at the 12 sampled hospitals, who were working on the day of distribution of questionnaires, and who agreed to participate in the study voluntarily were included in the study. There are two types of nurses in the Lao healthcare system, namely registered nurses, who have higher levels of vocational training (e.g., diploma in nursing) associated with more years of instruction, and practical nurses, who have a certificate of nursing only with a shorter period of training. For the purpose of this study, both registered and practical nurses were included in the sample.

The sample size was calculated using Power and Precision V4 software [38]. To achieve an effect size of 0.2 [6], a type one error of 0.05, and a power of 80%, a sample size of 458 was required. In order to mitigate the effect of missing data, however, we aimed to recruit 600 eligible participants into the study. In total, 595 agreed to participate, however, 36 did not return the completed questionnaire and one respondent’s questionnaire had to be discarded due to incomplete information. As a result, data of 558 participants were included in the analysis.

### Data collection

Data was collected in 2012, using a self-administered, structured questionnaire (Additional file 1).

The questionnaire included information on socio-demographic characteristics of the healthcare workers, and a HIV-related stigma scale consisting of 17 items and which was initially developed for healthcare providers in China [39]. In addition, HIV/AIDS knowledge was evaluated using a 10-item scale that has previously been used among health professionals in China [11]. The questionnaire was translated from English into Lao, and subsequently back-translated into English by a native speaker, to ensure semantic and content validity. Pre-testing of the questionnaire was undertaken with 40 healthcare professionals prior to data collection. Based on this, minor changes were made to some of the wording of items without changing the meaning. Prior to completing the questionnaire, all participants indicated their willingness to participate in the study by signing an informed consent form with their initials. To ensure confidentiality, identification numbers were used in place of personal names. No incentives or compensation for the time that participants took to compete the questionnaire was provided.

Prior to conducting the survey, two research assistants were provided one-day training on the study objectives and procedures, and on how to deal with potential problems that might arise during the survey. The research team was divided into two groups to distribute the self-administered questionnaire to the participants at the selected hospitals. Participants anonymously filled out the self-administered questionnaires and returned them to the principal investigator and research assistants.

### Dependent variable

The dependent variable (stigmatizing attitude) was measured by using the 17-item HIV-related stigma scale developed by Stein and Li [39]. The scale consists of five subscales: (1) discriminatory intent at work, (2) prejudiced attitudes, (3) internalized shame, (4) fear of PLWHA, and (5) opinion about healthcare for HIV/AIDS patients [39]. The subscale discriminatory intent at work consists of four items (items Q408-Q411) and assess respondents’ discriminatory actions while providing care to patients at their work places. The subscale prejudiced attitudes consists of four items (items Q401-Q404) that evaluate respondents’ feelings of prejudice toward PLWHA. The subscale internalized shame consists of three items (items Q415-Q417) and assess respondents’ feelings of shame in providing care to HIV/AIDS patients. Fear of AIDS, includes three items (items Q412-Q414) and measures a feelings of fear towards PLWHA. The subscale opinion about healthcare for HIV/AIDS patients, consists of three items (items Q405-Q407) and evaluates feelings of not being able to provide good care to HIV/AIDS patients. Each item in the scale was scored using a five-point Likert scale, ranging from (1) strongly agree to (5) strongly disagree, where, based on the scale guidelines, higher scores indicate higher levels of stigmatizing attitudes towards PLWHA [39]. In this study, the scale demonstrated good internal consistency with a Cronbach’s alpha score of 0.81.

### Independent variables

The independent variables included the socio-demographic characteristics of the healthcare workers, including sex, age, religion, ethnicity, education level, and marital status [40, 41]. Information related to participants’ professional characteristics included type and level of profession, institution of employment, number of years of professional experience in medical or nursing field, experience (duration) of giving care to PLWHA, and experience of formal HIV/AIDS training [30]. Other independent variables included the duration of providing care to PLWHA, and the number of HIV/AIDS cases encountered during their work experience [6, 30]. In addition, we assessed their HIV/AIDS knowledge using a 10-item scale which was previously used among health professionals in China (Li et al., 2007). Items were coded (1) for correct answers or (0) for incorrect answers or “unknown” responses. Results for the 10 items were totaled. Higher scores indicated higher general knowledge of HIV/AIDS. Scores equal to or exceeding 9 points, the median score, were defined as high knowledge scores.

### 2.5. Data analysis

Data entry and all statistical analyses were conducted using Stata/SE 11 [42]. Descriptive statistical analysis was used to describe the socio-demographic characteristics and the professional characteristics of respondents and scores on the HIV stigma scale. Comparisons between groups were performed by chi-square test and *t*-test. Bivariate and multiple linear regression analyses were carried out to examine the associations between stigmatizing attitudes and independent variables.

Before running the multiple linear regression models, the relationships between all continuous and dichotomous independent variables were examined using the Spearman rank correlation coefficient test to check for collinearity. High Spearman correlations were found between the following items: ‘age’ and ‘years of working experience in the medical professional’ (rs = 0.874); ‘ever provided care to PLWHA’ and ‘number of HIV/AIDS cases’ (rs = 0.966); and ‘ever provided care to PLWHA’ and ‘length of time provided care to HIV/AIDS patients’ (rs = 0.967). Furthermore, a high Spearman correlation was found between ‘number of HIV/AIDS cases encountered’ and ‘duration of providing care to PLWHA’ (rs = 0.946). Due to the multicollinearity in the data, three independent variables were removed from the multiple linear regression models, namely ‘age’, ‘ever provided care to PLWHA’, and ‘number of HIV/AIDS cases encountered’. *P*-value less than 0.05 was taken as the significance level for all analyses.

### Ethical considerations

The study was approved by the Research Ethics Committee of the Graduate School of Medicine of the University of Tokyo and by the Ethics Committee of the University of Health Sciences in Lao PDR. During the data collection, the completed questionnaires were kept in a locked filing cabinet at the Faculty of Postgraduate Studies, the University of Health Sciences, Vientiane, with the key held by the principal investigator. Subsequently, the completed questionnaires were taken to Japan and stored in a locked cabinet at the Department of Community and Global Health, Hongo Campus, the University of Tokyo.

## Results

### Socio-demographic characteristics of the participants

The socio-demographic characteristics of respondents are provided in Table 1. As seen, out of the 558 participating healthcare workers, 277 (49.7%) were doctors and 281 (50.3%) were nurses. The mean age of doctors was 39 years, standard deviation (SD) 9.9 SD and the mean age of nurses was 35 years, SD 9.8. Most of the respondents were female (66.3%) and, of these, 146 (52.7%) were doctors, compared to 131(47.3) doctors being male. There were also more female nurses than male nurses (224, 79.7% and 57, 20.3% respectively). The majority (83%) of doctors had an education level equal to, or higher than Bachelor degree, whereas only 10.3% of nurses had completed a Bachelor degree.

**Table 1:** Socio-demographic characteristics of participants

Variables	Total (*n* = 558)		Doctors (*n* = 277)		Nurses (*n* = 281)	
	n	(%)	n	(%)	n	(%)
Age (years)	(mean 37 ± 10.1 SD)		(mean 39 ± 9.9 SD)		(mean 35 ± 9.8 SD)	
20–29	160	(28.7)	55	(19.9)	105	(37.4)
30–39	159	(28.5)	84	(30.3)	75	(26.7)
40–49	166	(29.8)	88	(31.8)	78	(27.7)
50–59	66	(11.8)	43	(15.5)	23	(8.2)
60 or more	7	(1.2)	7	(2.5)	0	0
Gender						
Female	370	(66.3)	146	(52.7)	224	(79.7)
Male	188	(33.7)	131	(47.3)	57	(20.3)
Religion						
Christian	19	(3.4)	9	(3.2)	10	(3.6)
Buddhism	539	(96.6)	268	(96.8)	271	(96.4)
Ethnicity						
Highland people	22	(4.0)	13	(4.7)	9	(3.2)
Lowland people	536	(96.0)	264	(95.3)	272	(96.8)
Education level						
< Bachelor	299	(53.6)	47	(17.0)	252	(89.7)
≥ Bachelor	259	(46.4)	230	(83.0)	29	(10.3)
Marital status						
Single	168	(30.1)	73	(26.3)	95	(33.8)
Married	377	(67.6)	201	(72.6)	176	(62.6)
Divorce	8	(1.4)	2	(0.7)	6	(2.2)
Widow	5	(0.9)	1	(0.4)	4	(1.4)

### Professional characteristics and HIV/AIDS care-related characteristics of the participants

Table 2 shows the professional characteristics and HIV-related knowledge and attitudes of participants. Of the total number of participants, registered nurses, at 31.7%, constituted the largest of the four professional groups (general doctors, physician specialists, registered nurses, and practical nurses). Most of the participants (66.1%) were working in referral hospitals (Table 2). Compared to doctors, a significantly higher proportion of nurses were working in referral hospitals (72.2% vs. 59.9%, *p* = 0.002). Less than half of the respondents had provided care to HIV/AIDS patients, with only 17.9% providing care to HIV/AIDS patients for more than three years (47, 17% of doctors and 53, 18.9% of nurses).

**Table 2:** The professional characteristics and HIV-related knowledge and attitudes of participants

Variables	Total (*n* = 558)		Doctors (*n* = 277)		Nurses (*n* = 281)		*p-*value
	n	(%)	n	(%)	n	(%)	
Type of service provider							
General doctor	159	(28.5)	159	(57.4)			
Physician specialist	118	(21.2)	118	(42.6)			
Practical nurse	104	(18.6)			104	(37.0)	
Registered nurse	177	(31.7)			177	(63.0)	
Institution of practice							
Non referral hospital	189	(33.9)	111	(40.1)	78	(27.8)	0.002
Referral hospital	369	(66.1)	166	(59.9)	203	(72.2)	
Year of work experience in medical professional							
1–5 years	188	(33.7)	88	(31.8)	100	(35.6)	0.614
6–15 years	159	(28.5)	80	(28.9)	79	(28.1)	
16 years or longer	211	(37.8)	109	(39.3)	102	(36.3)	
Provide care to HIV/AIDS patients							
No	333	(59.7)	164	(59.2)	169	(60.1)	0.822
Yes	225	(40.3)	113	(40.8)	112	(39.9)	
Length of time that provide care to HIV/AIDS patients							
Non	333	(59.7)	164	(59.2)	169	(60.1)	0.671
≤ 3 years	125	(22.4)	66	(23.8)	59	(21.0)	
> 3 years	100	(17.9)	47	(17.0)	53	(18.9)	
Number of HIV/AIDS cases encountered							
None	333	(59.7)	164	(59.2)	169	(60.1)	0.955
≤ 3 cases	108	(19.3)	55	(19.9)	53	(18.9)	
> 3 cases	117	(21.0)	58	(20.9)	59	(21.0)	
Training for HIV/AIDS							
No	295	(52.9)	147	(53.1)	148	(52.7)	0.925
Yes	263	(47.1)	130	(46.9)	133	(47.3)	
HIV/AIDS knowledge (mean 8 ± 1.5 SD, median 9)							
Low (score 0–4)	7	(1.3)	2	(0.7)	5	(1.8)	0.245
Medium (score 5–8)	226	(40.5)	120	(43.3)	106	(37.7)	
High (score ≥ 9)	325	(58.2)	155	(56.0)	170	(60.5)	
Stigmatized attitudes (mean 38 ± 7.9 SD, median 39)							
Low (score ≤ 39)	303	(54.3)	153	(55.2)	150	(53.4)	0.660
High (score > 39)	255	(45.7)	124	(44.8)	131	(46.6)	

In terms of HIV-related training and knowledge, just under half (47.1%) of participants had attended formal HIV training, and 155 (56%) of doctors and 170 (60.5%) of nurses demonstrated a high level of knowledge on the HIV knowledge scale. Regarding stigmatizing attitudes, 45.7% of all participants showed high levels of stigmatizing attitudes towards PLWHA (44.8% of doctors and 46.6% of nurses).

### Factors associated with stigmatizing attitudes of doctors and nurses towards PLWHA (whole scale)

The results of bivariate linear regression analysis among doctors are shown in Table 3. They show that several factors were found to be significantly associated with HIV/AIDS stigmatizing attitudes and discriminatory intent. Doctors who had provided care to HIV patients (Coef. = −2.47, 95% confidence interval [CI]: −4.29 – -0.66, *p* = 0.008), who had provided care to HIV/AIDS patients for a longer duration (Coef. = −0.11, 95% CI:-0.19 – -0.04, *p* = 0.003), and who had higher HIV/AIDS knowledge (Coef. = − 0.90, 95% CI:-1.54 – -0.27, *p* = 0.005) were less likely to show stigmatizing attitudes towards PLWHA, as measured on the HIV-related stigma scale. Multiple linear regression analysis revealed that doctors who had provided care to PLWHA for a longer duration and those who had higher levels of HIV/AIDS knowledge were less likely to demonstrate stigmatizing attitudes towards PLWHA (Coef. = −0.09, 95% CI:-0.17 – -0.02, *p* = 0.013 and Coef. = − 0.69, 95% CI:-1.34 – -0.04, *p* = 0.036 respectively).

**Table 3:** Factors associated with stigmatized attitudes (whole scale) of doctors and nurses towards PLHA (bivariate and multiple linear regression analysis)

Characteristics	Bivariate			Multivariate		
	Coef.	95% CI	*p*-value	Coef.	95% CI	*p*-value
Doctors (*n* = 277)						
Age*	0.04	−0.04 – 0.13	0.357	-	-	-
Gender (Male)	1.12	−0.68 – 2.92	0.222	0.50	−1.35 – 2.35	0.595
Religion (Buddhism)	2.22	−2.85 – 7.31	0.389	1.27	−3.72 – 6.28	0.615
Ethnicity (Lowland people)	0.79	−3.47 – 5.05	0.716	−0.75	−6.97 – 5.46	0.811
Education level (≥ Bachelor)	−2.17	−4.56 – 0.21	0.075	−1.44	−3.84 – 0.94	0.235
Type of health care service provider (Physician specialist)	0.56	−1.25 – 2.39	0.543	1.50	−0.38 – 3.39	0.118
Institution of practice (Referral hospital)	−1.45	−3.28 – 0.38	0.121	−1.03	−3.13 – 1.07	0.336
Year of working experience in medical professional	0.42	−0.65 – 1.49	0.440	−0.07	−0.81 – 0.65	0.830
Provide care to HIV/AIDS patients* (Yes)	−2.47	−4.29 – -0.66	0.008	-	-	-
Length of time that provide care to HIV/AIDS patients (100 days)	−0.11	−0.19 – -0.04	0.003	−0.09	−0.17 – -0.02	0.013
Number of HIV/AIDS cases encountered*	−0.005	−0.015 – -0.003	0.223	-	-	-
Training for HIV/AIDS (Yes)	−1.01	−2.81 – 0.79	0.270	−0.31	−2.18 – 1.54	0.736
HIV/AIDS knowledge	−0.90	−1.54 – -0.27	0.005	−0.69	−1.34 – -0.04	0.036
Nurses (*n* = 281)						
Age*	−0.06	−0.16 – 0.03	0.181	-	-	-
Gender (Male)	0.86	−1.54 – 3.27	0.481	−0.13	−2.58 – 2.30	0.910
Religion (Buddhism)	0.19	−5.04 – 5.44	0.941	1.90	−4.74 – 8.56	0.572
Ethnicity (Lowland people)	−1.22	−6.74 – 4.28	0.662	−1.04	−6.44 – 4.35	0.703
Education level (≥ Bachelor)	−1.35	−4.54 – 1.83	0.405	−0.47	−3.69 – 2.73	0.769
Type of health care service provider (Registered nurse)	−2.84	−4.83 – -0.86	0.005	−2.26	−4.25 – -0.27	0.026
Institution of practice (Referral hospital)	−0.26	−2.43 – 1.90	0.810	0.82	−1.37 – 3.02	0.460
Year of working experience in medical professional	−0.59	−1.74 – 0.54	0.303	−0.04	−0.85 – 0.77	0.920
Provide care to HIV/AIDS patients* (Yes)	−2.70	−4.66 – -0.74	0.007	-	-	-
Length of time that provide care to HIV/AIDS patients (100 days)	−0.11	−0.18 – -0.04	0.001	−0.09	−0.16 – -0.02	0.017
Number of HIV/AIDS cases encountered*	−0.01	−0.017 – -0.004	0.001	-	-	-
Training for HIV/AIDS (Yes)	−2.30	−4.23 – -0.37	0.019	−1.33	−3.31 – 0.64	0.187
HIV/AIDS knowledge	−0.47	−1.11 – 0.16	0.145	−0.24	−0.88 – 0.39	0.455

* Independent variables of: age, provide care to HIV/AIDS patients and number of HIV/AIDS cases encountered were not put in the model for multivariate analysis because of multicollinearity

Similar results were found for nurses. Nurses who had provided care to HIV patients (Coef. = −2.70, 95% CI:-4.66 – -0.74, *p* = 0.007), and for a longer duration (Coef. = −0.11, 95% CI:-0.18 – -0.04, *p* = 0.001), who had encountered more HIV/AIDS cases (Coef. = −0.01, 95% CI:-0.017 – -0.004, *p* = 0.001), and had received HIV/AIDS training (Coef. = −2.30, 95% CI:-4.23 – -0.37, *p* = 0.019) were less likely to report stigmatizing attitudes towards PLWHA. Multiple linear regression analysis revealed that nurses who had provided care to PLWHA for a longer duration were less likely to manifest stigmatizing attitudes (Coef. = − 0.09, 95% CI:-0.16 – -0.02, *p* = 0.017) and that registered nurses were less likely than practical nurses, to exhibit stigmatizing attitudes towards PLWHA (Table 3).

### Factors associated with stigmatizing attitudes of doctors and nurses towards PLWHA (subscales)

#### Discriminatory attitudes

Table 4 shows that doctors who had cared for PLWHA patients (Coef. = − 1.05, *p* < 0.001), had more years of experience in providing care to PLWA (Coef. = − 0.04, *p* < 0.001), had received HIV/AIDS training and had higher level of HIV/AIDS knowledge (Coef. = −0.62, *p* = 0.018 and Coef. = −0.16, *p* = 0.045 respectively) were less likely to have discriminatory attitudes towards PLWHA. The only statistically significant difference observed with regard to nurses, was that compared to practical nurses, registered nurses were less likely to have discriminatory attitudes (Coef. = − 0.61, *p* = 0.018).

**Table 4:** Factors associated with stigmatized attitudes (subscales) of doctors and nurses towards PLHA (bivariate linear regression analysis)

Characteristics	Discrimination intent at work		Prejudiced attitudes		Internalized shame		Fear of AIDS		No Good care for HIV patients	
	Coef.	*p*-value	Coef.	*p*-value	Coef.	*p*-value	Coef.	*p*-value	Coef.	*p*-value
Doctors (*n* = 277)										
Age	0.01	0.112	0.003	0,827	0.02	0.049	0.003	0.810	−0.01	0.344
Gender (Male)	0.25	0.286	−0.31	0.364	0.58	0.035	0.16	0.565	0.44	0.053
Religion (Buddhism)	0.98	0.141	−0.27	0.774	0.68	0.380	1.15	0.139	−0.31	0.625
Ethnicity (Lowland people)	0.65	0.241	−0.41	0.608	0.65	0.313	0.49	0.454	−0.59	0.268
Education level (≥ Bachelor)	0.14	0.655	−1.07	0.018	−0.69	0.057	−0.27	0.460	−0.26	0.376
Type of health care service provider (Physician specialist)	0.28	0.227	−0.42	0.222	0.46	0.097	0.12	0.668	0.11	0.609
Institution of practice (Referral hospital)	0.01	0.966	−0.95	0.006	−0.45	0.107	0.31	0.269	−0.36	0.119
Year of work experience in medical professional	0.13	0.150	−0.11	0.402	0.16	0.110	0.05	0.614	−0.14	0.106
Provide care to HIV/AIDS patients (Yes)	−1.05	<0.001	−0.34	0.317	−0.35	0.200	−0.49	0.082	−0.22	0.331
Length of time that provide care to HIV/AIDS patients (100 days)	−0.04	<0.001	−0.009	0.510	−0.02	0.094	−0.03	0.014	−0.02	0.028
Number of HIV/AIDS cases encountered	−0.001	0.204	0.001	0.569	−0.002	0.049	−0.001	0.270	−0.0008	0.469
Training for HIV/AIDS (Yes)	−0.62	0.008	−0.38	0.264	0.18	0.506	0.05	0.832	−0.24	0.280
HIV/AIDS knowledge	−0.16	0.045	−0.09	0.416	−0.27	0.004	−0.19	0.050	−0.16	0.038
Nurses (*n* = 281)										
Age	−0.01	0.468	−0.02	0.188	0.01	0.371	−0.02	0.188	−0.02	0.030
Gender (Male)	0.26	0.402	−0.91	0.047	0.53	0.161	0.64	0.091	0.33	0.257
Religion (Buddhism)	0.15	0.825	1.32	0.187	0.06	0.938	−0.76	0.358	−0.57	0.373
Ethnicity (Lowland people)	0.48	0.502	0.16	0.873	−0.23	0.788	−0.62	0.476	−1.02	0.132
Education level (≥ Bachelor)	0.03	0.935	−0.72	0.233	−0.19	0.692	0.005	0.991	−0.46	0.240
Type of health care service provider (Registered nurse)	−0.61	0.018	−0.32	0.394	−0.97	0.002	−0.78	0.014	−0.13	0.575
Institution of practice (Referral hospital)	0.05	0.843	0.07	0.852	−0.16	0.628	0.08	0.797	−0.32	0.230
Year of work experience in medical professional	−0.02	0.769	−0.15	0.276	0.11	0.349	−0.12	0.294	−0.21	0.021
Provide care to HIV/AIDS patients (Yes)	−0.25	0.315	−1.12	0.003	−0.56	0.070	−0.46	0.141	−0.29	0.232
Length of time that provide care to HIV/AIDS patients (100 days)	−0.01	0.144	−0.05	<0.001	−0.01	0.201	−0.02	0.095	−0.01	0.141
Number of HIV/AIDS cases encountered	−0.001	0.186	−0.004	0.001	−0.002	0.015	−0.002	0.003	−0.0004	0.569
Training for HIV/AIDS (Yes)	−0.42	0.095	−0.53	0.150	−0.48	0.113	−0.22	0.472	−0.64	0.007
HIV/AIDS knowledge	−0.13	0.105	0.07	0.565	−0.16	0.100	−0.15	0.118	−0.08	0.282

Multiple linear regression showed that doctors with more experience in years with HIV/AIDS patients (Coef. = − 0.03, *p* = 0.001) or with formal HIV/AIDS training, were less likely to show discriminatory intent at work (Coef. = − 0.51, *p* = 0.032). While compared to practical nurses, registered nurses were less likely to hold discriminatory attitudes, (Coef. = − 0.56, *p* = 0.031, Table 5).

**Table 5:** Factors associated with stigmatized attitudes (subscales) of doctors and nurses towards PLHA (Multiple linear regression analysis)

Characteristics	Discrimination intent at work		Prejudiced attitudes		Internalized shame		Fear of AIDS		No good care for HIV patients	
	Coef.	*p*-value	Coef.	*p*-value	Coef.	*p*-value	Coef.	*p*-value	Coef.	*p*-value
Doctors (*n* = 277)										
Gender (Male)	0.15	0.508	−0.37	0.268	0.32	0.246	0.16	0.546	0.22	0.342
Religion (Buddhism)	0.17	0.859	0.18	0.896	−0.15	0.888	1.15	0.138	0.56	0.548
Ethnicity (Lowland people)	0.63	0.239	−0.87	0.282	0.71	0.268	−0.24	0.797	−0.64	0.231
Education level (≥ Bachelor)	0.36	0.288	−0.81	0.110	−0.62	0.128	−0.44	0.276	−0.23	0.493
Type of health care service provider (Physician specialist)	0.43	0.062	0.30	0.444	0.86	0.005	0.008	0.978	0.39	0.114
Institution of practice (Referral hospital)	0.02	0.917	−0.79	0.040	−0.49	0.125	0.51	0.069	−0.49	0.056
Year of work experience in medical professional	0.16	0.058	−0.22	0.101	0.10	0.354	0.04	0.669	−0.17	0.049
Length of time that provide care to HIV/AIDS patients (100 days)	−0.03	0.001	0.001	0.903	−0.02	0.187	−0.03	0.020	−0.02	0.095
Training for HIV/AIDS (Yes)	−0.51	0.032	−0.32	0.346	0.30	0.272	0.32	0.258	−0.09	0.689
HIV/AIDS knowledge	−0.11	0.181	−0.04	0.711	−0.24	0.014	−0.16	0.090	−0.13	0.097
Nurses (*n* = 281)										
Gender (Male)	0.08	0.783	−1.09	0.015	0.41	0.281	0.55	0.149	0.09	0.741
Religion (Buddhism)	−0.13	0.881	1.40	0.147	0.10	0.923	−0.59	0.478	0.22	0.786
Ethnicity (Lowland people)	0.53	0.453	−1.39	0.280	−0.23	0.790	−0.13	0.901	−0.96	0.150
Education level (≥ Bachelor)	0.18	0.668	−0.54	0.358	−0.23	0.653	0.15	0.774	−0.008	0.984
Type of health care service provider (Registered nurse)	−0.56	0.031	−0.13	0.724	−0.92	0.003	−0.73	0.021	0.03	0.904
Institution of practice (Referral hospital)	0.25	0.389	0.34	0.397	0.16	0.649	0.27	0.428	−0.20	0.442
Year of work experience in medical professional	0.01	0.888	−0.04	0.781	0.13	0.234	−0.03	0.754	−0.18	0.042
Length of time that provide care to HIV/AIDS patients (100 days)	−0.008	0.398	−0.06	<0.001	−0.009	0.432	−0.01	0.234	−0.002	0.812
Training for HIV/AIDS (Yes)	−0.34	0.170	−0.24	0.523	−0.36	0.234	−0.01	0.957	−0.58	0.014
HIV/AIDS knowledge	−0.08	0.298	0.03	0.762	−0.08	0.394	−0.09	0.380	−0.04	0.553

NB: For independent variables such as: age, provide care to HIV/AIDS patients and number of HIV/AIDS cases encountered did not put in the model for multivariate analysis, because of multicollinearity

#### Prejudiced attitudes

As seen in Table 4, doctors educated to bachelor level or above (Coef. = −1.07, *p* = 0.018) and who worked at referral hospitals (Coef. = − 0.95, *p* = 0.006) were also less likely to have prejudiced attitudes relative to their less educated counterparts and those employed at non-referral hospitals. Factors associated with lower levels of prejudiced attitudes in nurses were providing care to PLWHA at least once (Coef. = −1.12, *p* = 0.003) and for longer durations (Coef. = − 0.05, *p* < 0.001), and exposure to more PLWHA (Coef. = − 0.004, *p* = 0.001). Male nurses were less likely than female nurses to have prejudiced attitudes towards PLWHA (Coef. = − 0.91, *p* = 0.047).

Multiple linear regression analysis revealed doctors working at referral (central) hospitals were less likely than those working at non-referral (regional/district) hospitals, to express prejudiced attitudes (Coef. = − 0.79, *p* = 0.040). Male nurses were less likely than their female counterparts to express prejudiced attitudes (Coef. = − 1.09, *p* = 0.015). In addition, nurses with more experience of working with HIV/AIDS patients were less likely to express prejudiced attitudes (Coef. = − 0.06, *p* <0.001).

#### Internalized shame

Male doctors (Coef. = 0.02, *p* = 0.049) and those of older age (Coef. = 0.58, *p* = 0.035), were more likely to exhibit internalized shame towards PLWHA than were female doctors and those of younger age. Other factors associated with doctors expressing lower level of internalized shame were more contact with HIV/AIDS cases (Coef. = − 0.002, *p* = 0.049) and had higher HIV/AIDS knowledge levels (Coef. = − 0.27, *p* = 0.004). In terms of nurses, factors associated with having less internalized shame were being a registered nurse (Coef. = −0.97, *p* = 0.002) and having come into contact with more PLWHA (Coef. = − 0.002, *p* = 0.015) as seen in Table 4.

Multiple linear regression analysis revealed doctors with higher levels of HIV/AIDS knowledge were less likely than those with lower levels, to have internalized shame regarding PLWHA (Coef. = − 0.24, *p* = 0.014) or to harbor internalized shame (Coef. = 0.86, *p* = 0.005) compared to physician specialists (Table 5). As seen in Table 5, registered nurses were less likely to have internalized shame (Coef. = − 0.92, *p* = 0.003) than practical nurses.

#### Fear of AIDS

Doctors with more years of experience in providing care to PLWHA patients being less likely than those with less experience, to exhibit fear towards PLWHA (Coef. = − 0.03, *p* = 0.014). As with feelings of internalized shame, factors associated with nurses having less fear of AIDS were being a registered nurses (Coef. = − 0.78, *p* = 0.014) and being exposed to more PLWHA (Coef. = − 0.002, *p* = 0.003; Table 4).

Multivariate analysis revealed that that doctors with longer experience with HIV/AIDS patients, were less likely to be fearful of HIV/AIDS (Coef. = −0.03, *p* = 0.020) than their less experienced colleagues and that registered nurses were less likely to be fearful of HIV/AIDS (Coef. = −0.73, *p* = 0.021) than practical nurses.

#### Good care for HIV patients

Doctors who had provided care to PLWHA for more years (Coef. = − 0.02, *p* = 0.028) and those who had higher HIV/AIDS knowledge levels (Coef. = − 0.16, *p* = 0.038) were less likely to have feelings of not providing good care for HIV/AIDS patients. For nurses, being older (Coef. = − 0.02, *p* = 0.030), having more years of working experience (Coef. = − 0.21, *p* = 0.021) and receiving HIV/AIDS training (Coef. = − 0.64, *p* = 0.007) were associated with lower levels of feelings of not providing good care (Table 4).

Multiple linear regression showed that doctors and nurses with longer years of working experience with HIV/AIDS patients were less likely than their less experienced counterparts to have feelings of not providing good care for those patients (Coef. = − 0.17, *p* = 0.049, Coef. = − 0.18, *p* = 0.042 respectively, Table 5). Furthermore, nurses with formal HIV/AIDS training were less likely to have feelings of not providing good care for HIV/AIDS patients (Coef. = −0.58, *p* = 0.014; Table 5).

## Discussion

This study is the first to report on HIV/AIDS-related stigmatization among healthcare workers in Lao PDR. Of concern, is just under half of participants had been provided with formal HIV training, and nearly 50% of doctors and nurses included in the study had high levels of stigmatizing attitudes towards PLWHA. These attitudes can contribute to missed opportunities for prevention, education, and treatment, and thereby undermine Lao PDR’s efforts to manage and prevent HIV. Across the different health professionals included in this study, lower levels of HIV/AIDS knowledge were associated with higher levels of stigmatizing attitudes towards PLWHA. Higher level of HIV/AIDS knowledge was associated with lower likelihood of internalized shame among doctors. These results were not consistent with the previous study in China [11], though they reflect the findings of previous studies from Nigeria and Belize [1, 6, 43].

This study suggested that stigmatizing attitudes, including discrimination at work, fear of AIDS, and prejudice, among doctors and nurses are less likely with longer periods of experience in treating PLWHA. Doctors, for example, who worked at referral (central) hospitals, where HIV/AIDS care wards have been established, had lower levels of prejudiced attitudes towards PLWHA than those who worked at regional and district hospitals. The assumption is, that through working with PLWHA, healthcare personnel gain more experience and familiarity with HIV/AIDS and, thus, acquire greater willingness to provide better care to HIV/AIDS patients. This finding was similar to that of a study from India [31]. Given these doctors were also working at central level facilities, it may also be that there was a higher level of institutional support and resources to manage HIV/AIDS patients [11]. Institutional level support should include gloves for invasive procedures, sharps containers, soap and water or disinfectant for handwashing, and post-exposure prophylaxis, in case of work-related potential exposure to HIV. It should also include making sure relevant policies, handwashing procedures or other critical information in key areas in the healthcare setting are visible to support health workers [10]. Health workers being able to protect themselves through universal precautions is important not only in preventing patient to health worker transmission but also in reducing health worker stigma and discrimination towards PLWHA due to fear of causal contact [10].

Physician specialists also showed greater feelings of shame, than did general doctors, stemming from their work in caring for PLWHA in the present study. Since physician specialists may feel that they have more knowledge and higher social standing than general doctors, they might be prone to feeling more shame in interacting with PLWHA [30]. In addition, among nurses, male nurses reported a lower level of prejudiced attitudes than did female nurses. The association could stem from the male-dominated nature of the Lao PDR, as a result of which, women are typically held to a higher moral standard than men and is consistent with studies in China [11] and Belize [6]. It may also relate to the highest prevalence of HIV being in high risk populations, such as sex workers [44], who are mainly perceived to be women and often experience stigmatization, compounded by the criminalization of act of selling sex in the Lao PDR [32].

HIV-related stigma within healthcare settings has been attributed to misperceptions surrounding transmission, a review conducted by Chambers and colleagues [24], however, highlighted how emotions also contribute to HIV-related stigma. Studies undertaken in Nigeria also suggest that medical or scientific education related to HIV is unlikely on its own, to be sufficient in terms of changing practices, and that attitudes and cultural beliefs also need to be addressed [45–48]. This study illustrates that prejudice can also be present in practitioners despite them having a level of understanding about HIV transmission. It supports other research that has highlighted the need for HIV knowledge and awareness raising interventions to take into account not only people’s knowledge needs, but to also acknowledge and address affective aspects as decision making [24]. This relates to the moral aspects of stigma, and the interconnections between moral attributions and the devaluation of certain social groups which, in turn, reinforce the status quo and serve to maintain dominant social values and constructions of what is right and good [24, 49]. Addressing HIV stigma in healthcare workers, therefore, requires a holistic approach that addresses knowledge and fear, creates an enabling environment for the consistent implementation of universal precautions (including sterile rubber gloves, working autoclaves, and access to free HIV testing for providers), and addresses the social and emotional aspects that shape stigmatizing attitudes and practices an increase willingness to work with PLWHA [11, 24, 50]. This may also explain, at least in part, why doctors working at the central level referral hospitals with HIV/AIDS care wards had lower levels of prejudiced attitudes towards PLWHA, as these providers are more likely to have had the capacity to implement universal precautions.

This study had several limitations that should be taken into account in interpreting the results. Firstly, the study used a convenience sampling method to select participants, as a result of which the results might not be generalizable to all healthcare workers and may have led to an under or over-representation of some groups of health workers. Given those who worked at referral hospitals, where HIV/AIDS care wards have been established, had lower levels of prejudiced attitudes towards PLWHA than those working in district hospitals, excluding five district hospitals due to size, means there may be some under reporting of negative attitudes. Secondly, as the issues dealt with were of a sensitive nature, there is a risk of social desirability bias in the doctors’ and nurses’ responses and thus stigmatizing attitudes could be under reported. A self-administered questionnaire, however, was used, by which every participant was free to respond to the questions privately, thus minimizing the risk of such bias. Another limitation of the study is the questionnaire did not include questions related to knowledge of or perceived capacity to implement universal precautions, which can influence attitudes to working with PLWHA. Despite such limitations, this study is the first study in Lao PDR to assess the stigmatizing attitudes of doctors and nurses towards PLWHA. The results provide information to policy-makers and health facility administrators, suggesting the need for continued education about HIV to health professionals.

## Conclusion

This study reveals that health professionals in Vientiane, Lao PDR, often hold stigmatizing attitudes towards PLWHA, and this has been observed elsewhere in low HIV prevalence settings. The study potentially provides a baseline against which interventions could be designed, implemented and evaluated. Given the relatively low levels of formal HIV training and the association between low levels of knowledge and stigmatizing attitudes, addressing the knowledge needs at the individual level is likely to be an important first step in reversing these negative attitudes. Integrating HIV knowledge into pre- and in-service training of doctors and nurses could help to address this. Training should include knowledge of HIV transmission and the application of universal precautions [51]. It should also include building an understanding of what stigma is, how it manifests and the harmful health effects of stigma on individuals, families, the community and the healthcare system [51]. It is also important that education programs address the shame and blame directed at PLWHA, by providing all healthcare workers opportunities to reflect on the underlying values that lead to the shame and blame and to assist them in delinking PLWHA with practices and professions, such as sex work, that are often considered immoral in Lao society. In a low prevalence country such as Lao PDR, exposure of health professionals to PLWHA may be relatively low. Yet, stigmatizing attitudes and prejudice were lower among doctors and nurses with longer periods of experience in treating PLWHA. Given this, including PLWHA in training sessions to provide, with prior training and support, testimonials and co-facilitation can help health workers understand the pernicious effect of stigma [10]. On-going monitoring of healthcare worker attitudes and behavior towards individuals with HIV/AIDS is also important, and can be used to evaluate change. Findings from such evaluations can also be fed back to health workers, as a way of helping to build a culture where stigmatizing attitudes are not tolerated.

In addition to interventions at the individual and healthcare facility level, consideration should be given to decriminalizing sex work and amending the current HIV Law which, under Article 69, makes it a criminal offence for a person to deliberately spread HIV infection to others [32]. Such laws can have stigmatizing effects and act as deterrence in accessing HIV services [32]. Progressively increasing access to HIV healthcare services at all levels, including ART, is also important in terms of reducing stigma, as well as providing access to all of those who need it. Ultimately, investing in effective HIV-stigma reduction interventions is important not only from an individual perspective, but also to enhance the uptake of early HIV testing and treatment compliance, given their important role in preventing further HIV transmission.

## Additional file

Additional file 1: Questionnaire for doctors’ and nurses’ views on people living with HIV/AIDS. Questionnaire for doctors and nurses views on people living with HIV/AIDS. (DOCX 29 kb)

## Abbreviations

AIDS: acquired immune deficiency syndrome
ART: antiretroviral therapy
HIV: Human immunodeficiency virus
PDR: People's Democratic Republic
PLWHA: People living with HIV/AIDS
