# Supplementary material for: An investigation of stigmatizing attitudes towards people living with HIV/AIDS by doctors and nurses in Vientiane, Lao PDR
Source: BMC Health Serv Res. 2017 Feb 10;17:125. doi: 10.1186/s12913-017-2068-8 (PMC5301416; doi:10.1186/s12913-017-2068-8)
Supplement: Additional file 1: — Questionnaire for doctors’ and nurses’ views on people living with HIV/AIDS. Questionnaire for doctors and nurses views on people living with HIV/AIDS. (DOCX 29 kb) [file 12913_2017_2068_MOESM1_ESM.docx]

| ID |  |
| --- | --- |

**QUESTIONNAIRE**

**Questionnaire for doctors and nurses’ views on people living with HIV/AIDS**

Remarks: The objective of this questionnaire is to collect information about knowledge, attitudes and practices toward people living with HIV/AIDS (PLWHA) among doctors and nurses. This is confidencial, so we do not record your name. This questionnaire will be destroyed after the answers are recorded to the computer. To investigate the information, please answer all questions to the best of your ability.

1. **Socio-demographic characteristics of the participant**

| N0 | Question | Answer | Remark | Code |
| --- | --- | --- | --- | --- |
| Q101 | What is your age? (Year) | --------------------- |  |  |
| Q102 | What is your gender? (check one) | 1. Male  2. Female |  | [ ] |
| Q103 | Religion: (check one) | 1. Buddhism  2. Christian  3. Muslim  4. Other: ____________ |  | [ ] |
| Q104 | Ethnicity: (check one) | 1. Lowland people  2. Midland people  3. Highland people |  | [ ] |
| Q105 | Education level: | 1. Certificate/diploma  2. Bachelor  3. Master  4. PhD  5. Post-graduate  6. Post-doctorate  7. Other: _____________ |  | [ ] |
| Q106 | Marital status | 1. Single  2. Married  3. Divorce  4. Widow |  | [ ] |

1. **Professional characteristics of the participant**

| N0 | Question | Answer | Remark | Code |
| --- | --- | --- | --- | --- |
| Q201 | Name of Institution where currently employed | -------------------------------------- |  |  |
| Q202 | Wich ward or department are you currently working | -------------------------------------- |  |  |
| Q203 | Profession: | 1. Doctor  2. Nurse |  | [ ] |
| Q204 | If doctor, in which field are you currently working? | 1. General doctor  2. Specialist  + please specify:..................... |  | [ ] |
| Q205 | If nurse, in which field are you currently working? | 1. Practical  2. Registered |  | [ ] |
| Q206 | Which of the following best describes your current status/title? | 1. I am a staff doctor  2. I am a staff nurse  3. I am a head of department  4. I am a head of nurses in the department |  | [ ] |
| Q207 | How many years of experience have you had as a medical professional? (Since the beginning of your work) | 1. 1 ~ 5 years  2. 6 ~ 10 years  3. 11 ~15 years  4. 16 ~ 20 years  5. 21 years or more |  | [ ] |
| Q208 | Have you ever given care to people living with HIV/AIDS as patients during your professional practice? | 1. Yes  2. No |  | [ ] |
| Q209 | If yes, how many year ? please specify | ................year, .............month |  | [ ] |
| Q210 | If yes, how many cases? please specify | .........................cases |  | [ ] |
| Q211 | If yes, where? | 1. in the hospital where you work 2. outside hospital  3. both |  | [ ] |
| Q212 | If outside hospital please state, where? | ----------------------------------- |  |  |
| Q213 | Have you ever had formal training or workshop on the care of people living with HIV/AIDS? | 1. Yes  2. No |  | [ ] |
| Q214 | If yes please state, where? | 1. within the country 2. international  3. both |  | [ ] |

**3. The following questions are to be answered “yes”, “no”, or “don’t know”. Please tick your responses and answer all questions. Please tick one.**

| N0 | Question | Answer | Remark | Code |
| --- | --- | --- | --- | --- |
| Q301 | Do you think that AIDS can be curable?  (Please tick one) | 1.Yes  2. No  3. Don’t know |  | [ ] |

| Q302 | Do you think that HIV can be transmitted through pregnancy? (Please tick one) | 1.Yes  2. No  3. Don’t know |  | [ ] |
| --- | --- | --- | --- | --- |
| Q303 | Do you think that HIV can be transmitted through childbirth? (Please tick one) | 1.Yes  2. No  3. Don’t know |  | [ ] |
| Q304 | Do you think that HIV can be transmitted through breast-feeding?  (Please tick one) | 1.Yes  2. No  3. Don’t know |  | [ ] |
| Q305 | Do you think that mosquitoes transmit HIV?  (Please tick one) | 1.Yes  2. No  3. Don’t know |  | [ ] |
| Q306 | Do you think that HIV can be transmitted through daily contact, such as sharing public toilet? (Please tick one) | 1.Yes  2. No  3. Don’t know |  | [ ] |
| Q307 | Do you think that HIV transmission can be stopped by more nutrient intake?  (Please tick one) | 1.Yes  2. No  3. Don’t know |  | [ ] |
| Q308 | Do you think that physical exercise can stop HIV transmission?  (Please tick one) | 1.Yes  2. No  3. Don’t know |  | [ ] |
| Q309 | Is an HIV vaccine already available?  (Please tick one) | 1.Yes  2. No  3. Don’t know |  | [ ] |
| Q310 | Do you think that patients with sexual transmitted disease are more likely to get HIV?  (Please tick one) | 1.Yes  2. No  3. Don’t know |  | [ ] |

**4. Please anwser the following questions as “strongly agree”, “agree”, “no opinion”, “disagree”, or “strongly disagree”. Please tick one.**

| N0 | | Question | Answer | Remark | Code |
| --- | --- | --- | --- | --- | --- |
| Q401 | | People who got infected with HIV through sex or drug use got what they deserved  (Please tick one) | 1. Strongly agree  2. Agree  3. No opinion  4. Disagree  5. Strongly disagree |  | [ ] |
| Q402 | | People who got infected with HIV through commercial sex activities deserve sympathy  (Please tick one) | 1. Strongly agree  2. Agree  3. No opinion  4. Disagree  5. Strongly disagree |  | [ ] |
| Q403 | | People who got infected with HIV through drug use deserve sympathy  (Please tick one) | 1. Strongly agree  2. Agree  3. No opinion  4. Disagree  5. Strongly disagree |  | [ ] |
| Q404 | | People who behave promiscuously should be blamed for AIDS  (Please tick one) | 1. Strongly agree  2. Agree  3. No opinion  4. Disagree  5. Strongly disagree |  | [ ] |
| Q405 | People who got infected with HIV/AIDS through commercial sex activities deserve good quality medical care  (Please tick one) | | 1. Strongly agree  2. Agree  3. No opinion  4. Disagree  5. Strongly disagree |  | [ ] |
| Q406 | People who got infected with HIV/AIDS through drug use deserve good quality medical care  (Please tick one) | | 1. Strongly agree  2. Agree  3. No opinion  4. Disagree  5. Strongly disagree |  | [ ] |
| Q407 | People who got infected with HIV/AIDS through blood donation deserve good quality medical care  (Please tick one) | | 1. Strongly agree  2. Agree  3. No opinion  4. Disagree  5. Strongly disagree |  | [ ] |
| Q408 | You would be willing to work with HIV positive patients  (Please tick one) | | 1. Strongly agree  2. Agree  3. No opinion  4. Disagree  5. Strongly disagree |  | [ ] |
| Q409 | If you worked with HIV positive patients, you would provide the same quality of care to them that you provide to other patients (Please tick one) | | 1. Strongly agree  2. Agree  3. No opinion  4. Disagree  5. Strongly disagree |  | [ ] |
| Q410 | If the superior in your hospital asked you to do a physical examination of a known HIV positive patient, you would be willing to do so  (Please tick one) | | 1. Strongly agree  2. Agree  3. No opinion  4. Disagree  5. Strongly disagree |  | [ ] |
|  |  | |  |  |  |
| Q411 | If you worked with HIV positive patients, you would interact with them just like other patients  (Please tick one) | | 1. Strongly agree  2. Agree  3. No opinion  4. Disagree  5. Strongly disagree |  | [ ] |
| Q412 | You feel afraid of PLWHA  (Please tick one) | | 1. Strongly agree  2. Agree  3. No opinion  4. Disagree  5. Strongly disagree |  | [ ] |
| Q413 | You would not buy from a food vendor who has HIV/AIDS  (Please tick one) | | 1. Strongly agree  2. Agree  3. No opinion  4. Disagree  5. Strongly disagree |  | [ ] |
| Q414 | You would not share eating utensils with a PLWHA because you are afraid of HIV infection.  (Please tick one) | | 1. Strongly agree  2. Agree  3. No opinion  4. Disagree  5. Strongly disagree |  | [ ] |
| Q415 | If you worked with HIV positive patients, you would wish that you could change your job so that you would never have to deal with PLWHA (Please tick one) | | 1. Strongly agree  2. Agree  3. No opinion  4. Disagree  5. Strongly disagree |  | [ ] |
| Q416 | You would feel ashamed if someone you know got HIV/AIDS  (Please tick one) | | 1. Strongly agree  2. Agree  3. No opinion  4. Disagree  5. Strongly disagree |  | [ ] |
| Q417 | You would feel ashamed if someone in your family got HIV/AIDS  (Please tick one) | | 1. Strongly agree  2. Agree  3. No opinion  4. Disagree  5. Strongly disagree |  | [ ] |

Thank you very much for your time. The answers you have given us will provide information to

help improve health services to pleople living with HIV/AIDS.
